# Hospitalizations associated with endemic and non-endemic mosquito-borne arboviruses in Canada, 2002–2023

**DOI:** 10.1371/journal.pone.0347106

**Published:** 2026-04-13

**Authors:** Maria Elizabeth Mitri, Antoinette Ludwig, Joanne Tataryn, Salima Gasmi, Mandy Whitlock, Peter A. Buck, Annie-Claude Bourgeois

**Affiliations:** 1 Zoonoses Division, Centre for Foodborne, Environmental and Zoonotic Infectious Diseases (CFEZID), Public Health Agency of Canada (PHAC), Ottawa, Ontario, Canada; 2 National Microbiology Laboratory (NML), Public Health Agency of Canada, Saint-Hyacinthe, Quebec, Canada; Instituto Nacional de Salud Publica Centro de Investigaciones sobre Enfermedades Infecciosas, MEXICO

## Abstract

Mosquito-borne arboviruses pose a growing public health concern in Canada, particularly in the context of climate change and increased global travel. This study aimed to quantify the burden of endemic and non-endemic mosquito-transmitted arboviral diseases in Canada by examining hospitalization trends from 2002 to 2023. Using administrative hospital data from the Canadian Institute for Health Information (CIHI) and national West Nile virus (WNV) surveillance data, we analyzed patient demographics, temporal and spatial patterns, and disease classification. Hospitalizations were classified as endemic (e.g., WNV) or non-endemic (e.g., dengue, chikungunya, Zika, yellow fever) based on historical presence and vector establishment in Canada. A total of 2,470 unique hospitalizations were identified, with 56.4% attributed to endemic diseases and 39.7% to non-endemic diseases. WNV accounted for over 99% of endemic-related hospitalizations, with peaks in 2003, 2007, and 2012 aligning with national surveillance data. Hospitalizations were highest among males aged 75–79 years, particularly in the southern regions of Saskatchewan, Manitoba, and Ontario. Non-endemic disease hospitalizations, primarily due to dengue and chikungunya, increased after 2010 and were more evenly distributed throughout the year, reflecting travel patterns. Younger adults (20–49 years) were most affected. The study highlights limitations in diagnostic coding and surveillance coverage, particularly the exclusion of Quebec data and underreporting of emerging arboviruses. These findings underscore the utility of hospital administrative data in complementing traditional surveillance systems and identifying populations at risk for severe outcomes. As climate change and travel continue to influence arboviral disease dynamics, integrated data sources are essential for guiding public health planning and response.

## Introduction

Surveillance of mosquito-borne diseases in Canada is becoming increasingly more important in the context of climate change, as well as other anthropogenic factors that are shaping the future of these diseases [1]. Under warming conditions, increased mosquito-transmitted disease activity is expected, including the introduction and expansion of competent vectors, increasing the risk of sporadic, localized non-endemic disease outbreaks and the potential for these diseases to become endemic [2]. For diseases already endemic in Canada, this may create greater unpredictability or variability in the geographic distribution and seasonality of these diseases [3]. The establishment and maintenance of surveillance systems for multiple diseases may not always be feasible due to various resource constraints, including available staff, costs and laboratory capacity. In order to monitor these diseases, while also creating sustainable surveillance practices, a focus on more severe indicators (i.e., hospitalizations/deaths) may be beneficial to better understand disease burden in the community and guide prevention and planning efforts.

West Nile virus (WNV) represents the largest burden of illness among mosquito-transmitted diseases endemic in Canada [4]. First discovered in Canada in 2001, in wild avian hosts and positive *Culex* mosquito pools in Southwestern Ontario, the first human cases emerged the following year in 2002 [5]. Over the last 20 years, reported human and non-human WNV activity has fluctuated dramatically. Over this period, local acquisition of WNV in humans has been documented across several jurisdictions in Canada, except for the Territories (Yukon, Northwest Territories and Nunavut) and Atlantic Provinces (New Brunswick, Prince Edward Island, Nova Scotia and Newfoundland and Labrador), with a larger geographic range found in animal populations such as wild birds and equines [6,7]. Approximately 20% of WNV human cases are symptomatic and less than one percent develop more severe neurological symptoms, presenting with encephalitis or meningitis, requiring more advanced care, such as hospitalization, and supportive treatment [8,9]. Although WNV in humans is a nationally notifiable disease and surveillance data have been collected since 2002, limited information on risk factors for severe illness, including hospitalizations is captured through the surveillance system as a result of limited public health follow-up and inconsistencies in reporting practices across provinces/territories since the establishment of this surveillance system [8,10].

For several other endemic mosquito-borne diseases (MBDs), including eastern equine encephalitis and California serogroup viruses (California encephalitis, LaCrosse, Jamestown Canyon and snowshoe hare virus), there is no formal national surveillance system in place which makes it challenging to quantify the burden of these diseases [2,11]. Additional data on these infections, such as administrative hospital data, particularly for patients who present with more severe encephalitis and neurological symptoms, is needed to better elucidate populations most at risk for these diseases, follow trends over time and understand overall burden to the healthcare system.

The burden of illness on Canadians and the healthcare system from travel-acquired non-endemic mosquito-transmitted diseases are not well quantified. Frequently reported mosquito-transmitted arboviral diseases in Canadians returning from travel, demonstrated in previous studies between 2012−2017, are dengue, chikungunya and Zika, and to a lesser extent, yellow fever and Japanese encephalitis [12,13]. *Aedes albopictus and Aedes aegypti* are among the known competent vectors for chikungunya virus, all four dengue virus serotypes, yellow fever virus and Zika virus; *Aedes albopictus* is considered established in southern Ontario since it’s re-occurring documentation beginning in 2002, with *Aedes aegypti* discovered for the first time in 2016−17 in close proximity [14]. Many of these non-endemic mosquito-transmitted diseases are not currently nationally notifiable, and while systems such as the Canadian Travel Medicine Network (CanTravNet), have been put in place to capture information on travel-acquired diseases, there remain important gaps in our knowledge of travel-related arboviral diseases in the Canadian context [15]. Alternative sources of data could be leveraged to address gaps in surveillance for diseases that impact the health of Canadians.

Administrative hospital data, which captures information on hospital admissions, are held by the Canadian Institute for Health Information (CIHI) and have been collected since 1998 to varying degrees in provinces and territories of Canada. This study leverages existing data sources (i.e., National WNV Surveillance System) and CIHI administrative data to explore severe outcomes of endemic and non-endemic mosquito-transmitted arboviruses in Canada. This includes determining patient characteristics and populations most at risk for severe disease and assessing the prevalence of these diseases spatially and temporally between 2002–2023. The goals of this study are to explore the use of administrative data alongside case-based public health surveillance data to: i) quantify the overall burden of severe mosquito-borne arboviral disease in the Canadian healthcare system, ii) describe who is most at-risk for developing severe outcomes attributed to mosquito-borne arboviral diseases based on socio-demographic and geographical risk, and iii) generate baseline analyses to better inform future trends in mosquito-borne arboviral diseases, particularly in the context of climate change and emerging/re-emerging diseases. The results may inform early detection and outbreak response, resource allocation and preparedness, and public communication and health promotion efforts.

## Methods

Mosquito-transmitted arboviruses of interest for this study were chosen using the following inclusion criteria: i) whether the disease is currently endemic and/or has been isolated and documented in Canada historically ii) whether the disease has had a significant impact on Canadian travellers, and iii) whether there is potential for the non-endemic disease to become endemic in Canada (i.e., through already detected or established competent vectors such as *Ae. aegypti* and *Ae. albopictus.* [12,16].

### Data sources

#### Administrative hospital data.

Data on hospitalizations used in this report were retrieved from the Discharge Abstract Database (DAD) held by CIHI and accessed from the Data Coordination and Access Program (DCAP) at Public Health Agency of Canada (PHAC) [17]. The DAD refers to a national database that records information on discharges, deaths, sign-outs and transfers from acute care facilities across Canada. Data are received directly from acute care facilities, or from their respective health/regional authority or ministry/department of health in nearly all provinces and territories. In Quebec, acute inpatient hospitalizations are submitted through a different process to CIHI by the Ministère de la santé et des services sociaux (MSSS) and facilities are not required to report to the DAD [17]. For this reason, this study excludes hospitalizations in Quebec.

The national standard for morbidity data reporting in Canada is the International Statistical Classification of Diseases and Related Health Problems, Tenth Revision, Canada (ICD-10-CA) for which the ICD-10-CA codes for each disease listed in Table 1 were referenced [18]. In 2001, the ICD-10-CA classifications were introduced with a phased implementation across Canada and adopted by all provinces and territories by 2006. Updates to ICD-10-CA are released by CIHI every three years [19–21]. The ICD-10-CA to ICD-9-CA Conversion Tables were used to convert the relevant codes in the DAD for years prior to 2006 [22]. The final list of codes used in this study, both for ICD-10-CA and ICD-9-CA versions, can be viewed in Table 1.

**Table 1:** International Classification of Disease (ICD-9-CA and ICD-10-CA) codes for mosquito-transmitted arboviruses.

Disease	ICD-10-CA	ICD-9-CA ^±^
Endemic		
West Nile	A92.3, A92.30, A92.31, A92.32, A92.39	N/A (066.3 other mosquito-transmitted disease/fever)
Eastern equine encephalitis	A83.2	062.2
Non-Endemic		
California encephalitis (includes La Crosse)	A83.5	062.5
St. Louis encephalitis	A83.3	062.3
Western equine encephalitis	A83.1	062.1
Zika	A92.5 (Congenital Zika, P35.4)	N/A (066.3 other mosquito-transmitted disease/fever)
Dengue	A97.0, A97.1, A97.2, A97.9. A90, A91	061 (065.4 other mosquito-transmitted hemorrhagic fever)
Chikungunya	A92.0	N/A (065.4 other mosquito-transmitted hemorrhagic fever)/ 066.3
Japanese encephalitis	A83.0	062.0
Yellow fever	A95.0 (sylvatic), A95.1 (urban), 95.9	060, 0601, 0609
Rift Valley fever	A92.4	N/A (066.3 other mosquito-transmitted disease/fever)
Australian encephalitis (Murray Valley) (includes Kunjin virus disease)	A83.4	062.4
Unspecified		
Other mosquito-transmitted viral fevers	A92.8	N/A (066.3 other mosquito-transmitted disease/fever)
Mosquito borne viral fever, unspecified	A92.9	066.3 (other mosquito-transmitted fever)
Other mosquito-transmitted viral encephalitis and mosquito-transmitted viral encephalitis, unspecified	A83.8, A83.9	062.9, 0628 (mosquito-transmitted viral encephalitis, unspecified)
Mosquito-transmitted haemorrhagic fever	N/A	065.4

±ICD-9-CA codes were used for earlier records submitted during the study period by NB, MB and other PTs that had not yet converted to ICD-10-CA. For more details, please refer to the CIHI ICD-10-CA Implementation Schedule here: https://www.cihi.ca/en/icd-10-cacci-implementation-schedule.

Data were extracted from the DAD using SAS Enterprise Guide (version 7.1) for the 2002−2023 calendar years, including key variables related to demographic, geographic and diagnostic information. All hospitalizations where a patient was admitted and subsequently diagnosed with at least one diagnosis among the ICD-9-CA/ICD-10-CA diagnosis codes listed in Table 1, were considered for inclusion in this study. Instead of restricting the number of hospitalizations to only include patients whose primary diagnosis was associated with these diseases, a broader approach was taken by also including hospitalizations that had these diseases listed as subsequent diagnoses (i.e., not their primary diagnosis). This approach was taken to account for the potential for initial misdiagnosis, particularly in the context of novel or emerging diseases, and individuals who may have had several other more severe complications/co-morbidities. In addition to diagnostic information, patient demographics and geographical information was also extracted in order to conduct analyses.

#### National WNV surveillance data.

Data from the National West Nile virus Surveillance System provided an opportunity to further validate and support spatial-temporal trends of severe WNV infections observed in hospitalized patients. These data are reported voluntarily to PHAC by provincial/territorial health ministries/agencies and Canadian Blood Services (CBS). Further information about the national WNV case definition can be found on Canada.ca [23]. West Nile virus cases (confirmed, probable and suspect) classified as having neurological manifestations, the most severe form of WNV infection, were included in this study. Given that this study was unable to include hospitalization data from Quebec, cases reported through the National WNV Surveillance System by Quebec were also excluded from the study.

### Classification of endemic versus non-endemic diseases

For the purposes of this study, a disease is classified as endemic where the presence of the pathogen has been established in Canada through consistent reports or detections of human and/or non-human cases (equines, mosquitoes, birds or other animal surveillance) in specific regions, as well as the establishment of competent vectors. Four of the important endemic to Canada arboviruses include West Nile virus, Jamestown Canyon virus (JCV) and snowshoe hare (SSH) virus (part of the California serogroup (CSG)), and eastern equine encephalitis [24–26]. There are currently no distinct ICD-10-CA codes for several of the CSG viruses, particularly JCV or SSH (Table 1). Many of these have caused epidemics and/or epizootics in parts of the United States or Canada in past decades [26]. While these infections are likely acquired locally within Canada, the possibility of travel-related acquisition cannot be ruled out entirely as many of these diseases are present globally.

Diseases classified as non-endemic in this study are defined as those where there has never been reported cases in humans acquired in Canada or pathogen detections via animal/vector surveillance, despite the presence of competent vectors. This includes arboviruses like dengue, chikungunya, Zika, Japanese encephalitis, Rift valley fever, Australian encephalitis and yellow fever. Where a disease has appeared sporadically or in isolated detections, but, unlike endemic disease, is not always present at a steady and predictable level, this was considered to be a non-endemic disease. This includes diseases such as St. Louis encephalitis and western equine encephalitis, where cases in humans have been acquired in Canada or there have been detections in animal or mosquito populations, but are rare and/or isolated to a single point in time or outbreak (Table 1) [27–29]. California encephalitis, which includes La Crosse virus, and is a member of the California serogroup of viruses, is not known to be currently present in Canada and therefore considered to be non-endemic [30].

Hospitalizations with a generic ICD-9-CA or ICD-10-CA diagnosis code, such as ‘other mosquito-transmitted viral fever’ (A92.8, 066.3), could not be further classified as an endemic or non-endemic disease and hence classified as unspecified in this analysis. In addition, a disease classification of unspecified was given when multiple diagnoses were listed for the same hospitalized patient (e.g., West Nile virus and dengue) and a disease classification of endemic or non-endemic was not feasible.

### Data cleaning, validation and de-duplication

Data cleaning and validation was conducted in R Studio (version 2022.02.0 + 443) to assess the extent of missing data and completeness of variables in the DAD dataset. Duplicate hospitalizations or re-admissions for the same infection were defined as two or more hospitalizations within one year (365 days) matched by encrypted personal identifier, gender, age and forward sortation area (FSA) of residence, and subsequently removed from analyses [31]. This timeline was based on a previous study that found symptoms associated with long-term sequalae of WNV infection and other viral encephalitis resolved in about 50% of cases at 12 months following illness onset, however other studies suggest long-term sequalae may persist for years [10,32–35]. Furthermore, patients who were admitted without an available encrypted personal identifier, but had matching age and gender, were removed from analyses altogether, as it was not possible to discern whether these were duplicates.

### Epidemiological analysis

In order to assess trends over time, descriptive analyses were conducted for hospitalized patients based of disease classification (endemic vs. non-endemic) and year of admission during the study period. Similarly, trends related to the seasonality of hospitalizations for both endemic and non-endemic diseases were analyzed by month of admission and further differentiated by disease. The number of hospitalizations by five-year age group was calculated, by sex and disease classification, as well as the age-specific rate. This was done by using the annual July 1^st^ population estimates, by age and sex, generated by Statistics Canada, excluding population counts in the province of Quebec [36]. Given the large burden of disease due to WNV in the Canadian context, and among endemic-associated mosquito-transmitted arboviruses, additional analyses were undertaken for hospitalizations associated with WNV. Specifically, these hospitalizations were compared against the Canadian national human case surveillance data by year, excluding cases reported by Quebec, to estimate the proportion of cases requiring hospitalization, and assist with the validation of spatial-temporal trends of severe WNV infections requiring hospitalization. To capture the geographical distribution of endemic hospitalizations geography was assigned using the patient’s forward sortation area (FSA) of residence associated with their health card number using ArcGIS Desktop (version 10.8). Residents of Quebec seeking hospitalization in a different province were excluded from this analysis.

## Results

### Overall hospitalizations

Between 2002 and 2023, a total of 2,753 hospitalizations related to mosquito-transmitted arboviruses were documented in the Discharge Abstract Database in Canada, excluding Quebec. Following the removal of duplicates (n = 302), 2,470 unique patient hospitalizations, were identified. Of these, 1,636 (66%), 528 (21%), and 192 (~8%) hospitalizations had a recorded diagnosis of a mosquito-transmitted arbovirus among the first, second and/or third diagnoses, respectively.

There were 1,394 hospitalizations that occurred over the study period attributed to diseases that are endemic in Canada, representing 56.4% of all hospitalizations. There were 981 hospitalizations that occurred over the study period attributed to diseases non-endemic to Canada, representing 39.7% of all hospitalizations. The remaining 3.9% (n = 95) hospitalizations could not be further differentiated as endemic or non-endemic; 61 were assigned non-specific ICD-9-CA or ICD-10-CA codes pertaining to mosquito-transmitted arboviruses and 34 had both endemic and non-endemic mosquito-transmitted arboviruses listed (Table 2).

**Table 2:** Number and overall proportion of hospitalizations and deaths associated with mosquito-transmitted arboviruses, by disease, in Canada* between 2002-2023 in the Discharge Abstract Database (DAD).

Disease	Number of Hospitalizations (n)	Percentage of Overall Hospitalizations (%)	Number of Deaths (n)	Percentage of Deaths by Disease (%)
Endemic (n = 1394, 56.4%)				
West Nile	1393	56.4%	97	6.9%
Eastern equine encephalitis	1	<0.1%	0	n/a
Non-Endemic (n = 981, 39.7%)				
Dengue	831	33.6%	7	<1%
Chikungunya	100	4.1%	1	1.1%
Yellow fever	15	0.6%	2	9.1%
Japanese encephalitis	10	0.4%	1	11.1%
St. Louis encephalitis	7	0.3%	1	14.2%
California encephalitis	7	0.3%	0	n/a
Western equine encephalitis	4	0.2%	0	n/a
Zika (including congenital Zika)	4	0.2%	0	n/a
Rift Valley fever	2	<0.1%	1	50%
Australian encephalitis	1	<0.1%	1	100%
Unspecified (n = 95, 3.9%)				
Multiple MBD diagnoses^±^	34	1.4%	0	n/a
Mosquito-transmitted disease encephalitis (unspecified)	31	1.3%	4	10%
Mosquito-transmitted disease fever (unspecified)	30	1.2%	0	n/a
Total (All Hospitalizations)	**2470**	**100%**	115	**4.7%**

± Represents hospitalizations that had more than one disease diagnosis listed above. In this disease category, 27/34 had a diagnosis of both chikungunya and dengue.

*Excluding the province of Quebec

Overall, the majority of hospitalizations were diagnosed with WNV (56.4%), followed by dengue (33.6%) and chikungunya (4.1%) (Table 2). Deaths occurred in approximately 5% of all persons hospitalized, with deaths being reported in nearly 7% of individuals hospitalized for WNV (Table 2). While hospitalizations associated with St. Louis encephalitis, Japanese encephalitis and Australian encephalitis appeared to have a higher case fatality ratio in hospital, the small sample sizes reflected in the dataset makes these findings difficult to interpret (Table 2).

### Hospitalizations in Canada related to endemic mosquito-transmitted arboviruses

#### Trends over time.

**Fig 1:**
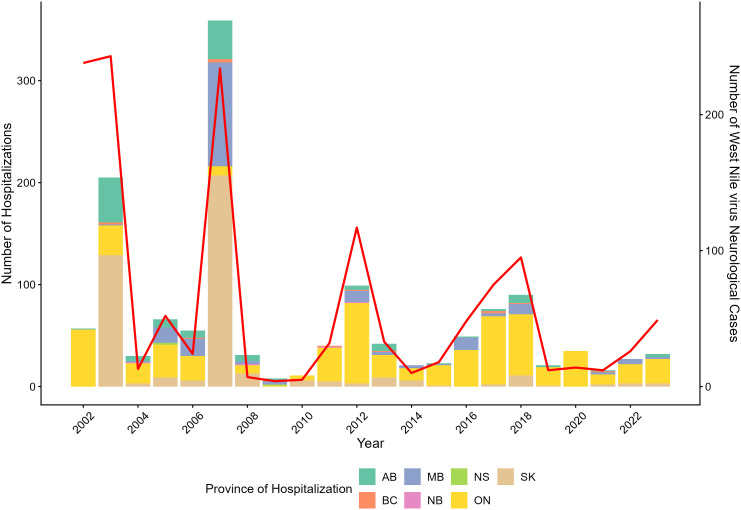
WNV associated hospitalizations and surveillance cases, by year and province. Annual crude number of West Nile virus associated hospitalizations in the Discharge Abstract Database (DAD) (n = 1,393) by year and province (coloured bars), compared with the National WNV Surveillance System reported neurological cases (red line), in Canada* between, 2002–2023. *Excluding the province of Quebec.

The annual number of hospitalizations for endemic mosquito-transmitted diseases ranged between 8–359 throughout the study period, with a mean of 63 hospitalizations annually. The largest number of hospitalizations occurred in 2007 (n = 359), 2003 (n = 205) and 2012 (n = 99). The incidence rates were also highest in these years; 1.42, 0.85 and 0.37 per 100, 000 Canadian population, respectively. In 2003 and 2007, Saskatchewan made up 62.9% (n = 129) and 57.7% (n = 207) of these hospitalizations, respectively. In all other years, Ontario made up the largest portion of hospitalizations (Fig 1).

**Fig 2:**
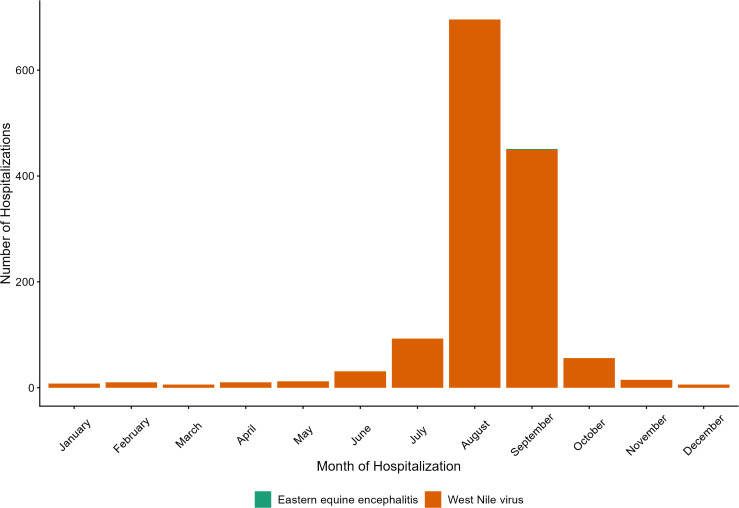
Seasonality of hospitalizations associated with endemic mosquito-borne arboviruses in Canada. Hospitalizations recorded in the Discharge Abstract Database (DAD) by month and disease, (n = 1,394), associated with endemic mosquito-transmitted arboviruses, in Canada*, 2002–2023. *Excluding the province of Quebec.

Of the 1,394 hospitalizations due to diseases classified as endemic in Canada, 1,393 (>99%) of these were hospitalizations associated with WNV (Fig 2). West Nile virus neurological cases (case-based surveillance), as reported by the National WNV Surveillance System, align with the number of hospitalizations observed during the study period, demonstrating a much greater number of cases than average (n = 62) for the years 2002, 2003, 2007, 2012, and 2018 (Fig 1).

The majority of hospitalizations associated with endemic arboviruses occurred in the month of August (n = 696, 49.9%), followed by September (n = 451, 32.4%) and July (n = 93, 6.7%) (Fig 2).

#### Geographic distribution.

**Fig 3:**
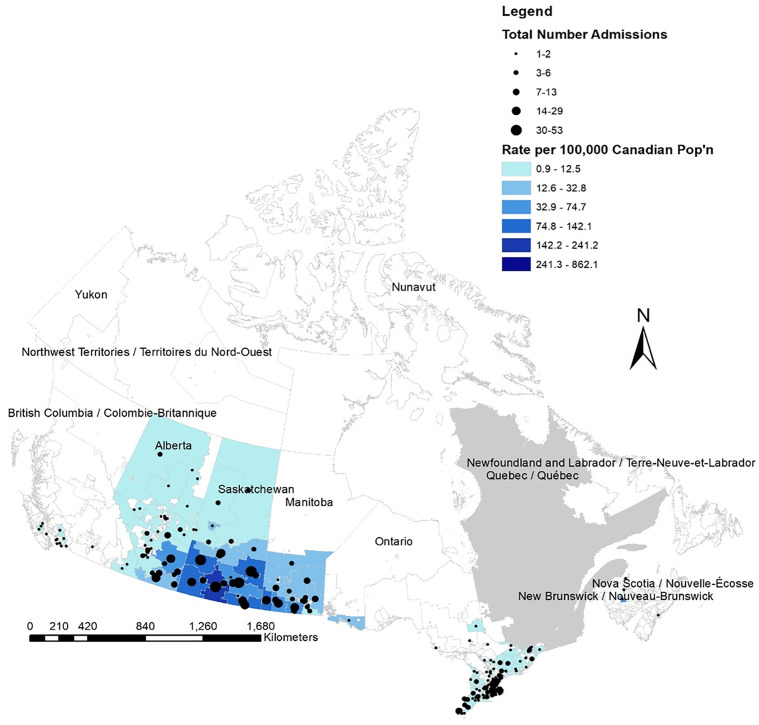
Map of hospitalizations associated with mosquito-borne arboviruses classified as endemic in Canada. Geographic distribution of hospitalizations in the Discharge Abstract Database (DAD), and cumulative incidence in Canada^*^, between 2002–2023, by Forward Sortation Area (FSA) of patient residence^±^, associated with endemic mosquito-transmitted arboviruses in Canada^*^, 2002–2023. * Excluding the province of Quebec. Quebec residents seeking hospitalization in another province/territory were also excluded from spatial analysis. ^±^Reflects patient residence according to health card and not necessarily associated with disease acquisition or where hospitalization occurred. **Source**: *Statistics Canada* © *Contains information copied with permission from Canada Post Corporation. Postal Code* ^*OM*^ *is an official mark of Canada Post Corporation. Contains information licensed under the Open Government Licence – Canada.* Forward Sortation Areas (FSA) Boundary File, 2021. *URL:* *https://www12.statcan.gc.ca/census-recensement/2021/geo/sip-pis/boundary-limites/index2021-eng.cfm?year=21. Adapted and produced using ArcGIS Pro version 10.08 by the author.*

Patient hospitalizations associated with endemic mosquito-transmitted arboviruses were observed in seven provinces: Ontario (44.4%), Saskatchewan (30.0%), Manitoba (13.9%), Alberta (10.0%), Nova Scotia (<1%) and New Brunswick (<1%). (Fig 3). The majority of hospitalizations during the study were concentrated in the southern regions of the provinces of Alberta, Saskatchewan, Manitoba, and Ontario based on the forward sortation area associated with health card (Fig 3).

#### Demographic characteristics.

**Fig 4:**
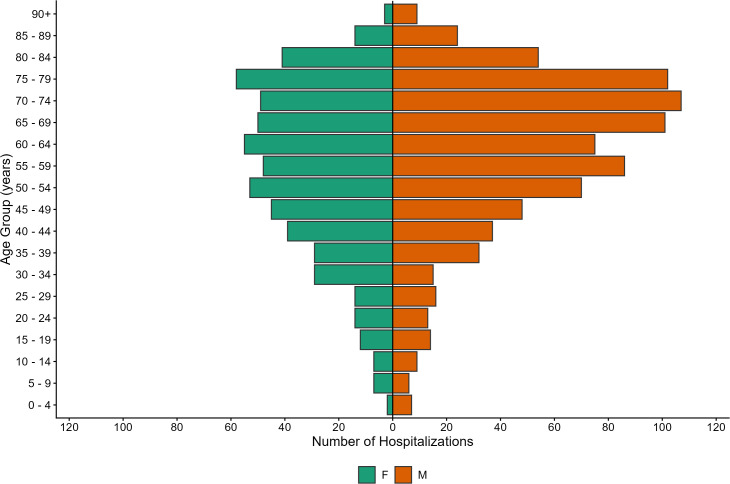
Demographic distribution of hospitalizations associated with endemic mosquito-borne arboviruses in Canada. Gender and age distribution of hospitalizations recorded in the Discharge Abstract Database (DAD) (n = 1,394), associated with endemic mosquito-transmitted arboviruses in Canada*, 2002–2023. *Excluding the province of Quebec.

Compared with females (n = 569, 40.8%), males were more represented (n = 825, 59.2%) among hospitalizations associated with endemic arboviruses (Fig 4). The cumulative age-specific incidence rate peaked in males between 75–79 years of age (32.0 per 100,000 population), which was approximately double the incidence in females in that same age group (15.4 per 100,000 population). Most (~75%) hospitalizations occurred between those aged 45–84 years of age, with the age-specific incidence rate peaking in those 70 years of age and older, among both sexes (Fig 4.

### Hospitalizations in Canada related to non-endemic mosquito-transmitted arboviruses

#### Trends over time.

**Fig 5:**
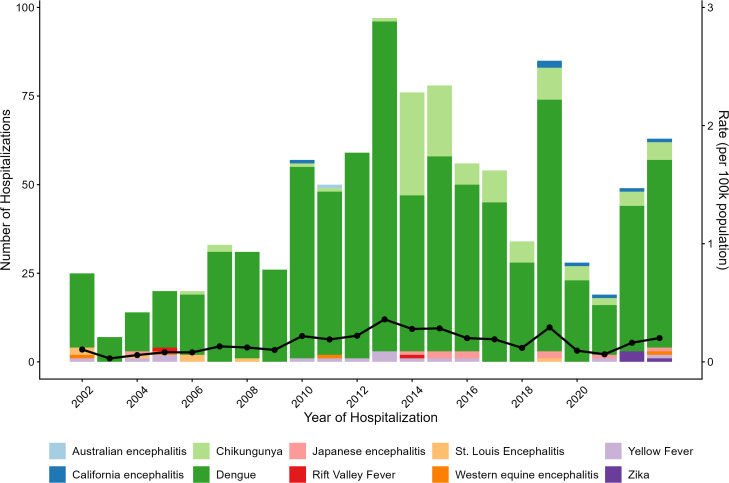
Annual hospitalizations associated with non-endemic mosquito-borne arboviruses in Canada. Annual crude number of hospitalizations (coloured bars) recorded in the Discharge Abstract Database (DAD) and rate (black line) (n = 981), associated with non-endemic mosquito-transmitted arboviruses in Canada, 2002–2023. *Excluding the province of Quebec.

During the study period, the number of hospitalizations that occurred due to diseases that are non-endemic in Canada ranged from 7–97 annually, with a mean of 45. Peak years of hospitalization occurred in 2013 (n = 97) and 2019 (n = 85) (Fig 5). Dengue made up the majority of hospitalizations, representing approximately 84.7% (n = 831/981) of all hospitalizations associated with non-endemic arboviruses and occurred throughout all years of the study period (Fig 5). Chikungunya represented 10.2% of hospitalizations (n = 100/981) and occurred in larger proportions in later years of the study period (2014 onwards). Other diseases, such as yellow fever and Japanese encephalitis were reported throughout multiple years in smaller proportions, and with no significant pattern (Fig 5).

**Fig 6:**
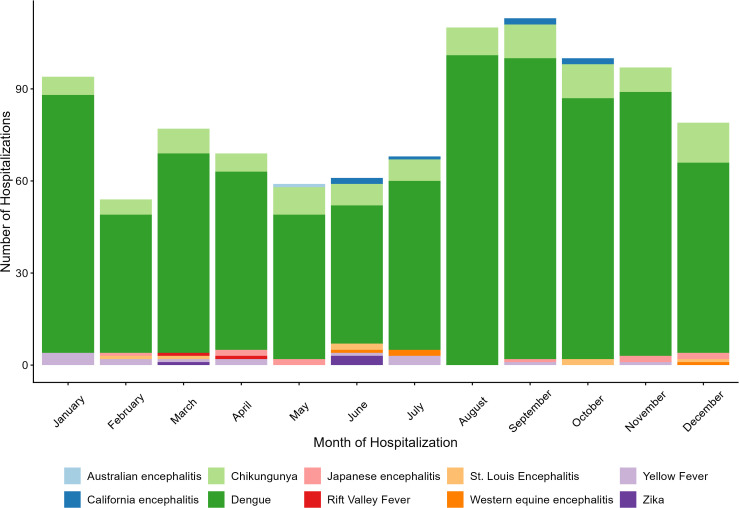
Seasonality of hospitalizations associated with non-endemic mosquito-borne arboviruses in Canada. Hospitalizations recorded in the Discharge Abstract Database (DAD) by month and disease (n = 981), associated with non-endemic mosquito-transmitted arboviruses in Canada*, 2002–2023. *Excluding the province of Quebec.

Compared with the seasonality of hospitalizations associated with endemic arboviruses, hospitalizations associated with non-endemic arboviruses occur more consistently throughout the year. The majority of hospitalizations associated with non-endemic arboviruses occurred in the month of September (n = 113, 11.5%), August (n = 110, 11.2%), October (n = 100, 10.2%), with the months between April – July recording a lower number of hospital admissions comparatively (Fig 6).

#### Demographic characteristics.

**Fig 7:**
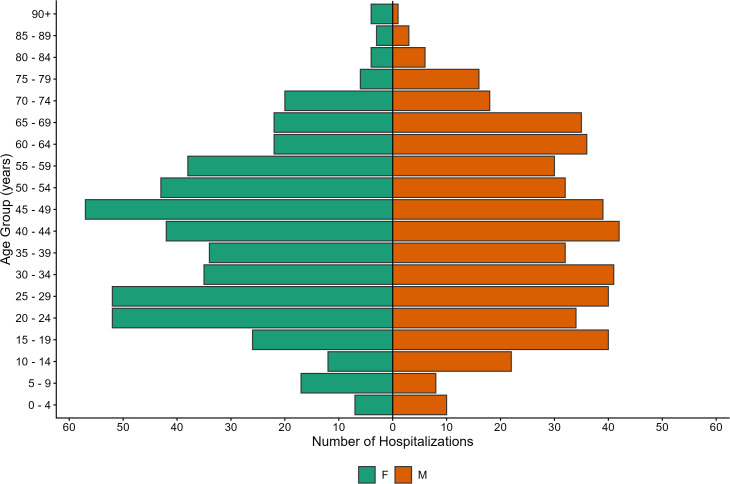
Demographic distribution of hospitalizations associated with non-endemic mosquito-borne arboviruses in Canada. Gender and age distribution of hospitalizations in the Discharge Abstract Database (n = 981), associated with non-endemic mosquito-transmitted arboviruses in Canada*, 2002–2023. *Excluding the province of Quebec.

Among hospitalizations associated with non-endemic arboviruses, males and females were equally distributed, with 496 females (50.6%) and 485 males (49.4%) represented (Fig 7). In addition, the incidence among males and females was relatively similar with males surpassing females slightly after 55 years of age. Hospitalizations associated with non-endemic arboviruses occurred more frequently in younger age groups, between those aged 20–49 years, when compared with hospitalizations associated with endemic arboviruses (Fig 4 and 7). Although relatively stable throughout all age groups, the age-specific incidence rate peaked in females aged 20–24 years (5.7 per 100, 000 population) and in males 65–69 years of age (5.9 per 100, 000 population). Conversely, rates were lower in males between 20–24 years of age (3.6 per 100,000 population), and females between 65–69 years of age (3.5 per 100, 000 population).

## Discussion

### Burden and trends of endemic mosquito-transmitted arboviruses in Canada

The majority of the hospitalizations (56.4%) recorded in the CIHI Discharge Abstract Database (DAD) during the study period, 2002–2023, included a diagnosis of a mosquito-transmitted arbovirus that is currently endemic in Canada. Of these hospitalizations, nearly all were associated with WNV (n = 1,393, 99.9%), contributing to the current evidence that WNV encompasses the largest burden of domestically acquired mosquito-transmitted disease since it was first detected in Canada in 2001 [37,38]. This also mirrors trends occurring in the United States, where the leading cause of domestically acquired arboviral disease is WNV, also the most common cause of epidemic viral encephalitis [39,40].

Results of this study showed that hospitalizations associated with WNV occurred in each year of the study period, with peaks observed in 2003, 2007, 2012, and 2018. This mirrored the pattern of WNV neurological cases reported through the National WNV Surveillance System, with outbreaks or peak case counts recorded in the same years, with the exception of earlier years (2002−03) [41]. Discrepancies between hospitalizations and cases reported through the National WNV Surveillance System occurred in 2002−2003, which may be attributed to limited clinical awareness in the early establishment of WNV in Canada resulting in misclassifications of ICD-9-CA codes at that time. It would also confirm that a considerable proportion of cases captured in the surveillance system did result in hospitalization, likely attributed to the severe outcomes associated with neurological manifestations of WNV infection. Studies have shown that WNV neurological cases are typically associated with more severe symptoms such as meningitis, encephalitis, and acute flaccid paralysis, and hospitalization rates have been estimated to be greater than 85% across all age groups among WNV cases with neurological syndrome [42]. During the outbreak years of 2003 and 2007, a large number of hospitalizations were represented by the western provinces of Alberta, Saskatchewan and Manitoba, while Ontario was proportionally more represented in the subsequent outbreak years of 2012, 2017 and 2018, similar to what is captured by the National WNV Surveillance System [41]. In 2020, hospitalizations were nearly double what was reported through national surveillance which may have been impacted by the COVID-19 pandemic due to limited public health capacity.

Trends in the seasonality of hospitalizations demonstrated that the majority of hospitalizations for endemic-associated arboviruses occurred during the summer months in Canada, between July and September, with the majority of hospitalizations occurring in August and September. This coincides with the peak in mosquito activity (specifically the peak of *Culex spp*, the main vectors of WNV) across Canada, as well as has been observed via the National WNV Surveillance System [5,43,44]. The hospitalizations occurring outside of the mosquito season suggest that some endemic-associated hospitalizations may have been acquired in other endemic areas outside of Canada and/or that long-term sequalae of these infections meant patients sought out hospital care months after initial infection. In addition to WNV, a single hospitalization of eastern equine encephalitis was identified during the study period in 2020.

Older age groups comprised a significant proportion of hospitalizations related to endemic-mosquito-borne arboviruses, with rates peaking between 70–79 years of age and were nearly double for males versus females. These elderly age groups are at increased risk for neurologic manifestations attributed to mosquito-transmitted viruses and are more likely to experience long-term sequalae of WNV illness [45]. This was supported by a study of WNV-associated hospitalizations, where WNV patients experiencing encephalitis were older and more likely to experience long-term mental and physical sequalae, as well a more severe hospital outcome [46]. Finally, the geographic distribution of endemic-associated hospitalizations, driven in large part by WNV-associated hospitalizations, demonstrated rates were highest among patients living in southern regions of Saskatchewan, Manitoba, and Ontario, which is supported by the spatial distribution of cases reported through the National WNV Surveillance System [41].

### Emerging challenges in identifying and tracking non-WNV arboviruses

Previous documentation of bunyaviruses, including the California serogroup viruses, demonstrate that they may be contributing to significant morbidity during the mosquito transmission season in Canada, and that their geographic range and seasonal risk is more widespread than WNV [11]. However, we were unable to quantify the burden of these diseases in hospital as there is no formal ICD-10-CA to identify these admissions. Alternatively, hospitalizations attributed to the California serogroup of viruses may become classified under an unspecified mosquito-borne disease code (Table 2). Outbreaks due to these diseases are expected to occur in the future, which may give rise to increases in severe cases and hospitalizations of patients experiencing meningitis and encephalitis [47,48].

### Hospitalizations due to non-endemic arboviruses and travel-related risks

A fewer number of overall hospitalizations were attributed to mosquito-transmitted arboviruses not currently endemic in Canada (39.7%). However, unlike the year-to-year fluctuations observed for hospitalizations due to endemic mosquito-transmitted arboviruses, an increasing trend of hospitalizations, due to non-endemic mosquito-transmitted diseases, was observed throughout the study period, particularly after 2010. Hospitalizations decreased between 2020 and 2021, likely attributed to the decrease in travel activity, due to the COVID-19 pandemic and strict border restrictions in Canada during this time. A large number of these hospitalizations were driven by dengue and chikungunya, which have been detected with increased frequency over the last few decades globally [49]. Peak hospitalizations of non-endemic mosquito-transmitted arboviruses occurred between 2013−2015 and 2019 and coincide with reported outbreaks in several countries throughout these same years. This includes chikungunya outbreaks noted in 2014−15 in the Caribbean/Americas and in southeast Asia in 2018, global outbreaks of dengue reported across multiple years with 2019 marking an unprecedented peak of dengue cases spread across 129 countries [50–52]. An increase in travel- acquired cases was also observed in the United States during this time and it is predicted that the risk of dengue to travellers is expected to increase and remain one of the most common causes of fever associated with viral infection in returning travellers [53]. Dengue was reported as one of the most common causes of fever in returning Canadian travellers between 2011−2012 [13]. While Zika virus was included in this study, with outbreaks documented in 2015−16 in Latin America and over 500 laboratory-confirmed cases in Canada as of June 2017, only four admissions related to Zika, including congenital Zika, were identified in the DAD during the study period [54,55]. The majority of Zika virus cases are mild and self-limiting, and therefore hospitalizations are uncommon which may explain why few hospitalizations were documented with this diagnosis. Studies have shown that the majority of cases with neurological complications of Zika virus infection were due to Guillain-Barre syndrome (75%); however, this diagnosis was not included for the purpose of this study [56].

Hospitalizations associated with non-endemic mosquito-transmitted arboviruses occurred more consistently throughout the entire year, with increases in late summer and winter months, likely associated with when Canadians travel most frequently. Compared to hospitalizations associated with endemic arboviruses, younger age groups—particularly females aged 20–29 and 40–49—were more frequently hospitalized for non-endemic arboviruses, likely reflecting increased travel-related exposure and behaviours [13].

### Limitations in administrative health data and surveillance systems

It is important to note that the use of ICD-9-CA/10-CA codes to identify confirmed or probable cases of any given disease in hospital may be a limitation of this study for several reasons. First, the entry of the ICD-9-CA/ICD-10-CA codes are dependent upon the discretion of the practitioner entering them which may vary based on several factors including unclear physician documentation, misinterpretation of clinical data, outdated or insufficient codes, use of “other” or “unspecified” codes, particularly in the case of more rare and emerging diseases. For example, the lack of equivalent ICD-9-CA codes for many of the diseases captured in this study (e.g., West Nile virus, Rift Valley Fever) means that hospitalizations captured prior to 2006, before the implementation of ICD-10-CA system across Canada, may be less specific. Therefore, these diseases may be underestimated for these early years, which is reflected by the comparisons between WNV hospitalizations versus case-based surveillance, and may be captured under a less specific ICD-9-CA code during this time. For non-endemic diseases, such as SLE and WEEV that remain rare in North America, there is a possibility that these diagnoses are due to misclassifications. Furthermore, many of these more rare, non-endemic diseases included in this study have limited diagnostic capacity in Canada, with few laboratories across the country capable of conducting these tests [11]. Given that a laboratory diagnosis is not always needed to administer the appropriate treatment for these diseases and their associated symptoms, hospitalizations associated with these arboviruses may only become classified according to their main symptoms, and not the disease itself (i.e., meeting a clinical case definition) to guide clinical care in a timely and flexible manner. Therefore, the specificity and sensitivity around the use of ICD-CA codes to accurately identify true cases, may vary due to several factors. The lack of hospitalization data for the province of Quebec is a considerable limitation of this study, as Quebec reported a considerable number of endemic-associated mosquito-transmitted arboviruses, including WNV, during the study period and remains one of Canada’s largest provinces by population size [10].

## Conclusion

This study provides a comprehensive assessment of the burden of mosquito-transmitted arboviral diseases in Canada using hospital administrative data, offering valuable insights into populations most at risk for severe outcomes. The findings confirm that West Nile virus (WNV) remains the predominant cause of arboviral-related hospitalizations in Canada, disproportionately affecting older adults and males, particularly in the southern regions of Saskatchewan, Manitoba, and Ontario. These trends were consistent with national surveillance data, validating the utility of hospital data as a complementary source for monitoring disease burden.

In contrast, non-endemic arboviruses such as dengue and chikungunya showed increasing hospitalization trends post-2010, primarily affecting younger adults, and reflecting global outbreak patterns and travel-related exposures. While the overall number of hospitalizations due to non-endemic diseases was lower than endemic ones, their year-round occurrence and rising trend suggest a growing public health concern, especially in the context of climate change and increased international travel.

### Considerations for public health practice

Compared to hospitalizations for other infectious diseases, arboviral-related admissions are relatively rare but represent severe outcomes that can strain healthcare resources during outbreak years. The study also highlighted gaps in diagnostic and surveillance capacity, particularly for rare and emerging arboviruses, underscoring the need for improved laboratory capacity and standardized reporting. These findings highlight the potential value of targeted prevention strategies—particularly for older adults and males living in high-incidence regions during peak mosquito seasons. Enhanced travel health messaging is also warranted, especially during periods of global arboviral outbreaks. Additionally, better integration of hospital data into routine surveillance systems could improve the assessment of severe outcomes. As climate change continues to influence vector distributions and disease dynamics, leveraging multiple data sources will be essential for strengthening early detection and response, guiding public health preparedness, informing resource allocation, and improving risk communication.
